# The interplay of transition metals in ferroptosis and pyroptosis

**DOI:** 10.1186/s13008-024-00127-9

**Published:** 2024-08-03

**Authors:** Frantisek Vana, Zoltan Szabo, Michal Masarik, Monika Kratochvilova

**Affiliations:** 1 Department of Physiology, Faculty of Medicine, Masaryk University, Kamenice 5, Brno, CZ-625 00 Czech Republic; 2 Department of Pathological Physiology, Faculty of Medicine, Masaryk University, Kamenice 5, Brno, CZ-625 00 Czech Republic; 3 First Faculty of Medicine, BIOCEV, Charles University, Prumyslova 595, Vestec, CZ-252 50 Czech Republic; 4 Regional Centre for Applied Molecular Oncology, Masaryk Memorial Cancer Institute, Zluty kopec 7, Brno, 656 53 Czech Republic

**Keywords:** Ferroptosis, Pyroptosis, Transition metal, Cancer, Cardiovascular disease, Neurological disease

## Abstract

Cell death is one of the most important mechanisms of maintaining homeostasis in our body. Ferroptosis and pyroptosis are forms of necrosis-like cell death. These cell death modalities play key roles in the pathophysiology of cancer, cardiovascular, neurological diseases, and other pathologies. Transition metals are abundant group of elements in all living organisms. This paper presents a summary of ferroptosis and pyroptosis pathways and their connection to significant transition metals, namely zinc (Zn), copper (Cu), molybdenum (Mo), lead (Pb), cobalt (Co), iron (Fe), cadmium (Cd), nickel (Ni), mercury (Hg), uranium (U), platinum (Pt), and one crucial element, selenium (Se). Authors aim to summarize the up-to-date knowledge of this topic.

In this review, there are categorized and highlighted the most common patterns in the alterations of ferroptosis and pyroptosis by transition metals. Special attention is given to zinc since collected data support its dual nature of action in both ferroptosis and pyroptosis. All findings are presented together with a brief description of major biochemical pathways involving mentioned metals and are visualized in attached comprehensive figures.

This work concludes that the majority of disruptions in the studied metals’ homeostasis impacts cell fate, influencing both death and survival of cells in the complex system of altered pathways. Therefore, this summary opens up the space for further research.

**Graphical abstract:**

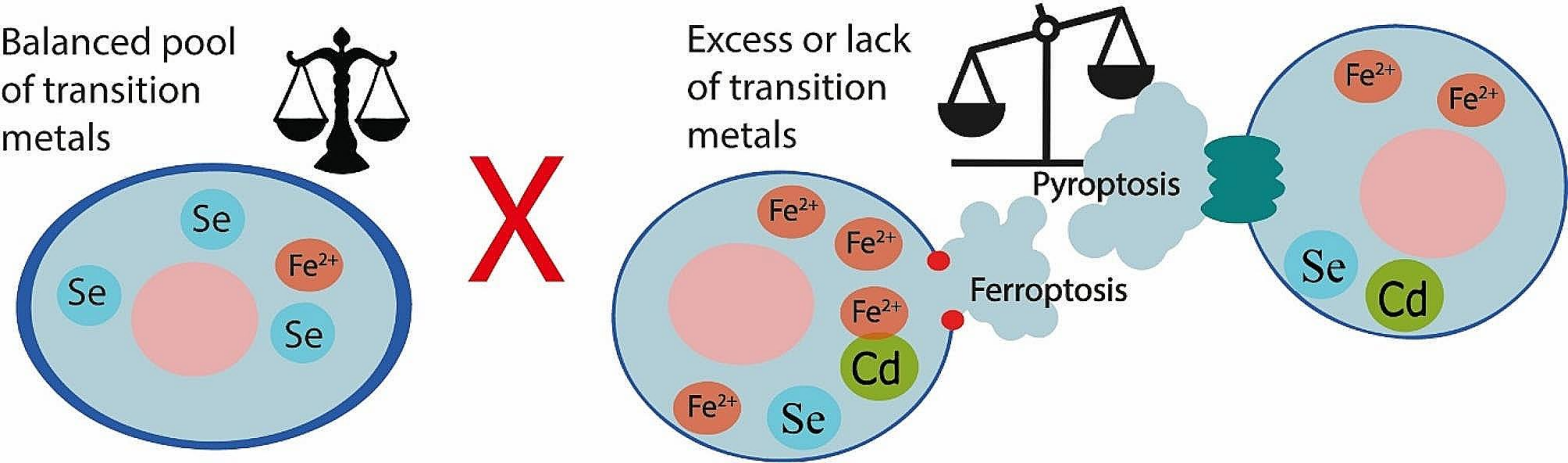

## Introduction

In this review, we aim to describe the role of transition metals in ferroptosis and pyroptosis. Ferroptosis and pyroptosis represent two distinct modes of regulated necrotic cell death, both of which have gained recognition in recent years. Given that cellular death plays a pivotal role in maintaining the homeostasis in our body, their importance is acknowledged across various research domains. These include embryology, evolution and developmental biology, various disease pathologies or novel therapy and drug discovery. Like all cellular processes, programmed cell death can be affected by external or internal factors.

One influential factor is the presence of transition metals, typically found in small quantities, making even minor fluctuations. They could be a critical determinant in the execution of ferroptotic and pyroptotic cell deaths. Consequently, these changes can have either beneficial or detrimental effects on the organism, depending on the specific circumstances.

We selected papers for this review based on their relevance to the topic, and therefore these studies have a great variability of experimental models (animals, cell lines). Because not all studies were performed on the same scientific model, some comparisons are not possible. Moreover, pyroptosis is type of cell death prominent only for cells of the immune system. However, this paper focuses on the resume of current state of knowledge.

Understanding the intricate relationships between cell death pathways and transition metals holds the potential to enhance our comprehension of health maintenance, disease prevention, and treatment. For example, modulating transition metal levels or targeting metal-related pathways can potentially be used to regulate these forms of cell death.

Today, the ever-growing importance of TMs (transition metals) in industrial usage means the possible exposure to these exotic materials in various forms. This opens another field for further research of the interactions yet unknown.

### Ferroptosis

#### Discovery of ferroptosis

**Fig. 1:**
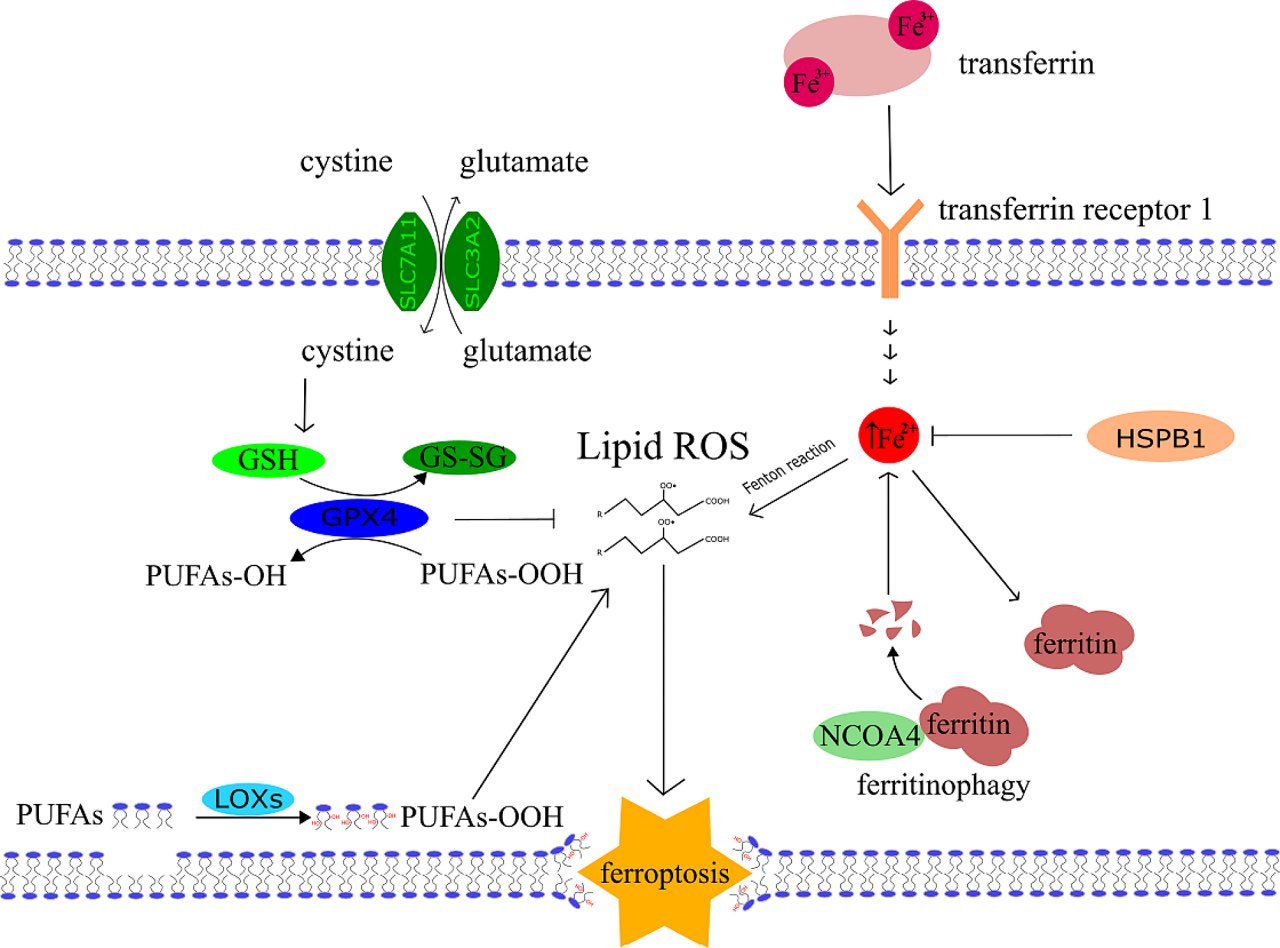
General mechanism of ferroptosis. Three main axes are described in this scheme. The first is an inhibitory axis centred around GPX4, which can convert lipid hydroperoxide into lipid alcohols. GPX4 needs glutathione (GSH) as a cofactor and its main component, cysteine, is delivered through the Xc-cystine-glutamate antiport system, which consists of two units SLC7A11 (Solute Carrier Family 7 Member 11) and SLC32A2 (Solute Carrier Family 32 Member 2). The second axis represents iron metabolism and the capability of iron to undergo the Fenton reaction, which is the main source of ROS reacting with lipids. Fe^2+^ reacts with hydroperoxide and generates the most potent oxygen radical, the hydroxyl radical. The third axis consists of lipoxygenases, and their substrates – PUFAs. Phosphorylated Heat shock protein 1 (HSPB1) acts as a negative regulator of ferroptosis by reducing cellular iron uptake and lipid ROS production [281]. Another factor elevating the Fe^2+^ pool is ferritinophagy, which is mediated by a selective cargo receptor Nuclear receptor coactivator 4 (NCOA4). The third axis is driven by lipoxygenases (LOXs) which allow enzymatic oxidation of PUFAs to form. PUFAs-OOH adding to the lipid ROS pool. All three mentioned axes increase the amount of lipid ROS in the membrane which then leads to its rupture and ferroptosis occurs

The initial research that caught attention of biomedicine scientists’ dates to 2003. In this study the ferroptosis-inducing substance erastin was recognised as an effective drug to kill tumourtransformed fibroblasts with the expression of oncoprotein K-Ras (Kirsten rat sarcoma virus) [1]. However, this early on, ferroptosis itself was not yet discovered. Ferroptosis is a recently defined necrosis-like cell death, first described by Dixon et al. in 2012 [2]. In their study, cell death was induced by erastin and inhibited by iron chelators, which underlines the crucial role of iron in this cell death mechanism [3, 4]. The cystine/glutamate transport system (Xc system) was further identified as a target of erastin [3, 4]. This system transports glutamate out of the cell in exchange for cystine, which is then used by the cell to synthesize glutathione (GSH, γ-Glutamylcysteinylglycine, non-protein thiol). Erastin treatment leads to the exhaustion of cellular GSH deposit and inhibits glutathione peroxidase 4 (GPX4). GPX4 prevents the peroxidation of lipids and usually downgrades the autoactivation of ferroptosis in the cell [3, 4]. The most important ferroptosis pathways are shown in the Fig. 1.

#### Execution of ferroptosis

Oxidative stress induces ferroptosis by the depletion of cellular antioxidative potential [5]. Reactive oxygen species (ROS) represent the driving force of oxidative stress [6]. The main source of ROS, the Fenton reaction, will be discussed further. Polyunsaturated fatty acids (PUFAs), found mainly in plasmatic membranes, undergo peroxidation due to oxidative stress [7]. This process leads to the rupture of the plasmatic membrane followed by osmolysis, which completes the ferroptotic death of cells [7].

#### Lipoxygenases and ferroptosis

Lipoxygenases (LOX) are enzymes that transform cholesterol and PUFAs (mainly arachidonic acid) found in cytoplasmatic membranes into hydroperoxide derivates and therefore contribute to lipid peroxidation [8]. Together with free radical-driven, iron-dependent Fenton reaction, the LOX activity creates the pool of lipid radicals, the hallmark of ferroptosis [9–11]. Lipoxygenases will also be further discussed in relation to iron and ferroptosis.

#### Mitochondria and ferroptosis

Typical morphological signs of ferroptosis are small mitochondria with condensed membranes and with the disappearance of the mitochondria cristae or with the rupture of the membranes [12]. Shrunken or ruptured mitochondria are one of the key morphological signs of occurring ferroptosis and therefore mitochondria are supposed to be related to ferroptosis [13]. The exact relationship is unclear since the depletion of mitochondria does not seem to have a clear effect on ferroptosis. In one study, the cells depleted of mitochondria could still undergo ferroptosis [14], while in another study authors described significantly attenuated ferroptosis [3]. Mitochondria have their own GPX4, but its inhibition does not typically induce strong lipid peroxidation in mitochondria and the ferroptosis driving lipid peroxidation appears mainly in the cytoplasm [15]. Mitochondria can contribute to ferroptosis in other ways, including ROS generation as a byproduct of oxidative phosphorylation. Specifically strong ROS generation is caused by electron leakage from respiratory chain complexes I and III. Such action produces hydrogen peroxide (H_2_O_2_) by the activity of superoxide dismutase (SOD)mediated dismutation [16]. H_2_O_2_ can then react with Fe^2+^ and produce hydroxyl radical which then reacts with the bis-allylic hydrogen in the structure of PUFAs and PUFA radicals are produced and can propagate [16]. Nevertheless, the detailed role of mitochondria in ferroptotic cell death is beyond the scope of this paper, see other authors review [12, 17].

#### Distinction between oxytosis and ferroptosis

In the absence of glutamine or during the inhibition of glutaminolysis the erastin-induced blockade of cystine import can lead to cell death. α-ketoglutarate, a product of glutaminolysis, can substitute the glutamine during the induction of ferroptosis [18]. High levels of glutamine in the extracellular fluid can induce ferroptosis via the inhibition of cystine uptake by the Xc- system [19]. This phenomenon was described in the cells of the central nervous system and was proven to be different from apoptosis and was given a name oxytosis [20]. Oxytosis has some similar features to ferroptosis, such as cysteine and GSH deprivation or ROS formation and lipid peroxidation, but differs in the terminal phase, which may be executed with the signs of apoptosis and is more dependent on the calcium than on iron [21].

#### The importance of ferroptosis in health and diseases

The physiological role of ferroptosis is not clear, but some studies suggest its importance during the development of the immune system, since the correct embryonal development of the immune system in humans depends on the adequate intake of PUFAs [22]. This was further supported by the study where T-lymphocytes without functional GPX4 were not capable of clonal expansion and did not protect against choriomeningitis virus or parasitical Leishmanias infection [23].

While ferroptosis has been linked to several diseases, it has been mainly studied in the context of cardiomyopathy [24], and in the context of neurological conditions, such as stroke, ischemia reperfusion injury or Parkinson´s, Huntington´s [25] and Alzheimer´s disease [4, 26, 27]. The occurrence in cardiomyopathy can be supported by the recent studies indicating that enhancing the glutathione system (the biochemical antioxidative system centred around glutathione [28]) and inhibiting ferroptosis, a cell death process intricately connected to the GSH system, holds a significant potential as a therapeutic approach for numerous cardiac diseases [29].

In Alzheimer´s disease, the role of ferroptosis is multimodal, but mainly connected to altered lipid metabolism and iron homeostasis. In experimental study on murine model, Bao and his team injected Amyloid Aβ into the brains of mice [30]. They noticed elevated levels of iron and ferritin in the hippocampus, along with decreased levels of GPX4. This finding shows one of the possible ways of ferroptosis involvement in the pathogenesis of Alzheimer´s disease.

Following cerebral ischemia, the blood-brain barrier (BBB) loses its tight junctional integrity, leading to disruption. This permits the entry of Fe^3+^ from the bloodstream into the brain parenchyma facilitated by transferrin (TF) and transferrin receptor (TFR) [31]. Fe^3+^ is subsequently reduced to Fe^2+^ and via the Fenton reaction generates ROS, promoting ferroptosis.

When reperfusion occurs after an ischemic period, excitatory amino acids represented by glutamate accumulate in the synaptic cristae. This leads to a decline in glutamate intake and an increase in the extracellular glutamate release, effectively inhibiting the Xc − system [32]. This all then enhances the ferroptosis during stroke ischemia and reperfusion in the brain [33].

Ferroptosis has been recognised as an important tumour-suppressing mechanism as well [34], and the evidence of its significance in oncological treatment is expanding each year [17]. All these information only highlight the importance of ferroptosis in health and disease. Detailed description would be topic large enough for another comprehensive review. However, a review on mechanisms of ferroptosis in related diseases has already been written by Feng, Tang et al. In this paper, authors discuss ferroptosis in great dept in each organ system [35].

### Pyroptosis

#### Discovery of pyroptosis

Pyroptosis is a modality of proinflammatory, regulated, necrotic-like cell death. Its cascade is centred around human Caspase-1, Caspase-4, and Caspase-5 or murine Caspase-11 [36]. They all belong to the family of proinflammatory Caspases and are essential for adequate reaction to pathogens [37]. The first observation of pyroptosis was during an experiment in which macrophages were infected with *Salmonella enterica* serovar Typhimurium (S. Typhimurium) or *Shigella flexneri* [38, 39]. During pyroptosis, proinflammatory Caspases (except for Caspase-12) activate on behalf of the inflammasome protein complex (see further) [40–42].

#### Tissue and cell specificity of pyroptosis

Not all cell types can undergo pyroptosis. This type of cell death is prominent for cells of the immune system, most notably macrophages, since canonical pyroptosis depends on the presence of inflammasomes [43]. Apart from immune system cells, pyroptosis has been documented in endothelial, neuronal cells, also in jejunal a renal epithelial cells and in various cancer cells (melanoma, osteosarcoma, colon carcinoma, lung cancer, triple negative breast cancer, cervical cancer, nasopharyngeal and oesophageal cancer cells) [11, 44–59].

#### Pyroptosis and imunity and inflammation

Pyroptosis has an important role in the activation of inflammation due to the release of the proinflammatory interleukins IL-1β and IL-18 [60]. This is accompanied by the release of DAMPs (damage-associated molecular patterns) [61] which enhance the stimulation of proinflammatory action, including the recruitment and activation of neutrophils, macrophages and other immune cells [62]. If the exposure to the proinflammatory factors is chronic, it can lead to the development of autoimmune diseases [63]. Some bacteria can replicate in macrophages in which pyroptosis is subsequently triggered. Pyroptosis leads to the termination of bacterial replication cycle and to the formation of PITs (pore-induced intracellular traps), that cage the bacteria in the fragments of the dead macrophage. Ligands of the PITs are recognised by neutrophils, triggering them to efferocytosis and to eliminate the pathogen [64].

#### Canonical and noncanonical pathways of pyroptosis

**Fig. 2:**
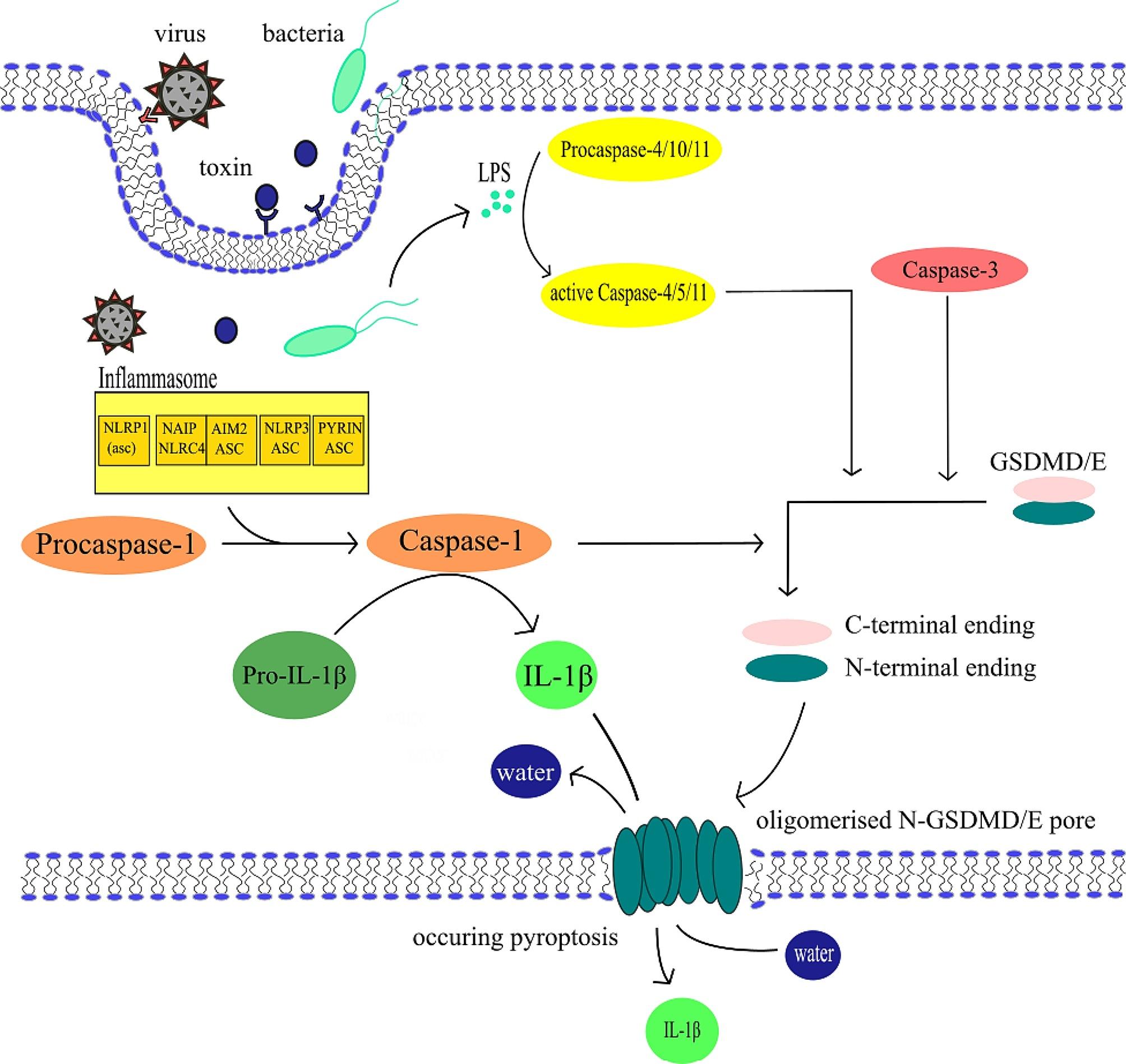
General pyroptosis mechanism – canonical and noncanonical pathway. Canonical pathway is centred around the inflammasome in this figure. Stimulation of this non-membranous organelle leads to the activation of Caspase-1, which then turns pro-IL-1β into IL-1β and cleaves gasdermins GSDME or GSDMD into N- and C-terminal ending. In membranes, the N‑terminal protein residues of GSDMs polymerise to form pores, causing water and electrolyte leakage and pyroptosis. IL-1β leaves the cell through these pores. Noncanonical pathway starts with LPS activating proCaspases − 4/-5/-10, which in their active forms are capable of cleaving gasdermins as well. During the activation of Caspase-3 the mechanism of cell death is decided by the expression level of GSDME. High levels of GSDME switch apoptosis to pyroptosis with oligomerised N‑GSDME pore formation [83]

Pyroptosis can be activated via mechanisms of canonical and noncanonical signalling pathway. The canonical pathway starts when DAMPs or PAMPs (pathogen associated molecular patterns) stimulate the formation of inflammasomes, which lead to the forming of pyroptosome and Caspase-1 activation [65]. The noncanonical pathway is induced by endotoxins produced by gramnegative bacteria (for example lipopolysaccharides (LPS) as portrayed in Fig. 2) [66]. Those endotoxins bind directly to the human proCaspase-4 [67]. In the murine model, PAMPs activate the proCaspase-11, homologous to the human proCaspase-4 and − 5 [66]. Both canonical and noncanonical pathways are visualised in Fig. 2. Based on evidence, it appears that the activation of specific proapoptotic Caspase cascades may be also required during pyroptosis [68]. This activation occurs due to BAX (bcl-2-like protein 4) activation, which leads to the permeabilization of the mitochondrial outer membrane [68]. Caspases activated during both canonical and noncanonical pathways cleave gasdermin D (GSDMD) [69–72]. The product of the cleavage, the N-terminal fragment of GSDMD, then oligomerizes in membranes to form pores causing necrotic-like cell death [73–76].

#### Gasdermins in pyroptosis

There are more gasdermins than gasdermin-D (GSDMD) and gasdermin-E (GSDME), that can participate in the activation of pyroptosis. Namely, gasdermin A (GSDMA) and gasdermin B (GSDMB) [77–79]. The cleavage of GSDME can be, in some situations, mediated by Caspase-3. Caspase-3 activators include certain chemotherapeutics, TNF-α (tumour necrosis factor) and Caspase-8, that usually would induce apoptosis. However, the Caspase-3/GSDME pathway can switch apoptosis to pyroptosis when the levels of GSDME are increased [80, 81]. This deviation is responsible for many side effects of certain chemotherapeutics [82], probably because of increased activation of pyroptosis in healthy cells. GSDME is typically expressed in healthy tissues, but tumour cells often lack GDSME [83]. On the other hand, in the absence of gasdermins, Caspase-1 can activate Caspase-3, Caspase-8 and Caspase-7 to start apoptosis, that serves as a reserve cell death mechanism in the case of pyroptosis insufficiency [84].

#### Inflammasomes

Inflammasomes are non-membranous organelles and are distinguished by the presence of receptors (inflammasome assembling PRRs – pattern recognition receptors) [80]. Such inflammasome assembling PRRs are expressed in many cell types, mainly in immune cells (macrophages, dendritic cells, neutrophils and epithelial cells) [85]. Innate immunity relies on inflammasomes, which are composed of intracellular sensors coupled with Caspase and interleukin activating systems [86]. Inflammasomes trigger pyroptosis and activate inflammatory cascades. NLRP3 (nucleotide-binding oligomerization domain-like receptor protein), NLRP6, NLRC4, and AIM2 (absent in melanoma) are prominent inflammasome members [86].

Two main types of inflammasomes were observed. Inflammasomes containing NOD-like receptors (NLR) and inflammasomes containing PYHIN (Pyrin and HIN domain) family members [87, 88]. NLR receptors are receptors containing nucleotide-binding oligomerizing domains [65]. This group of inflammasomes consists of NLRP3 (NLR family pyrin domain containing 3) based inflammasomes, inflammasomes based on NLRP1, and inflammasomes based on NLRC4 (NLR family CARD domaincontaining protein 4) [65]. It was observed, that other NLR proteins such as NLRP6 and NLRP7 can participate on the same process as well [65]. To the other (PYHIN) group of inflammasomes belong inflammasomes based on AIM2 (absent in melanoma 2) and IFI16 (interferoninducible protein 16) [65, 89].

ROS are essential inflammasome activating signals, activating inflammasomes through MAPK (mitogen activated protein kinase) and ERK1/2 (extracellular signal regulated kinase) [86]. Many of the transition metals have some relation to redox homeostasis and can be connected to pyroptosis with the repeating pattern of ROS activated pyroptosis [86]. Inflammasome dysregulation contributes to various diseases, including viral infections, silicosis, gout, and diabetes [90]. SIRT-1 (sirtuin-1) antioxidants can reduce inflammasome activation [90]. Inflammasomes and related pathways are potential therapeutic targets for various pathological conditions [86].

#### Pyroptosome

Pyroptosome is a large subcellular structure, which can be visualized via fluorescent staining of ASC (apoptosis associated speck-like protein containing a CARD, caspase activation and recruitment domain) [91]. It is important to note that pyroptosome is not necessary for the execution of pyroptosis, which can occur by NLRC4 dependent Caspase-1 activation [92]. Pyronecrosis, a pyroptosis subtype that can be triggered by *Shigella gonorrhoeae* or *Neisseria gonorrhoeae* is NLRP3 dependent and does not require activation by Caspase-1 [93, 94].

#### The importance of pyroptosis in health and diseases

As mentioned above, pyroptosis holds great importance in inflammation and immunity. Logically, any malfunction in the form of hyper- or hypo- activation can lead to various health problems. Especially among autoimmune diseases, we can distinguish NLR-3 related systemic autoinflammatory diseases (SAIDs).

The abnormal activation of inflammasomes is a significant factor driving SAIDs and hence pyroptosis may also contribute to SAIDs. The evidence indicates that the absence of GSDMD does not affect the activation of pro-IL-1β into IL-1β but rather hampers the release of IL-1β. This suggests that in SAIDs patients, the inflammatory response originates from excessive cytokine release via pores during pyroptosis [95]. Nevertheless, IL-1β can be released by other mechanisms than pyroptosis, and thus the connection between pyroptosis, IL-1β, and SAIDs does not need to be direct.

In some cases, the evidence for pyroptosis impacting the SAIDs is solid and supported by genetically distinct groups of SAIDs with relation to NLRP-3 and pyrin [96]. The list of NLRP-3 related SAIDs includes cryopyrin-associated periodic syndrome (CAPS), gout, and Crohn’s disease [97]. List of Pyrin associated SAIDs contains familial mediterranean fever (FMF), pyrin-associated auto-inflammation with neutrophilic dermatosis (PAAND) and livedoid ulcerative dermatitis [98–100].

Pyroptosis appears to play an important role in Covid-19 pneumonia, arthritis and other inflammatory diseases as well [101–104]. For other details on this topic, we recommend a review published in Nature [105].

Pyroptosis has importance in cancer biology as well, since immune system-mediated pro-cancer and anti-cancer mechanisms play a significant role in carcinogenesis. Pyroptosis can have a dual effect on tumours [106], it can suppress their occurrence and expansion, or on the other hand help with the development of a suitable microenvironment for tumour’s cell growth. The extended exposure to an inflammatory environment raises the risk of cancer development according to cell and tissue level theory of carcinogenesis. In particular, cytokines released during pyroptosis, such as IL-1β and IL-18, can promote tumour infiltration, thereby increasing the chances of tumorigenesis and metastasis [107, 108]. On the other hand, the anti-cancer activity of pyroptosis has not been fully understood yet and is still subject of research [106]. On the topic of pyroptosis’ dual role in cancer we recommend a great review published in Cellular & Molecular immunology journal [106].

### The importance of transition metals

Signalling is on any scale dependent on the location [109]. The medicinal compounds containing metals undergo metabolism to produce simple metal ions, which then interact with cellular components to generate novel compounds. These newly formed compounds may exhibit biological activity, thereby prolonging the therapeutic effects of the initial drug introduced into the system [110]. Thanks to this quality, they can contribute to signalling through coordinated spatial and temporal changes in their local concentrations and ligation statuses. These involve movements, redox changes or catalysis and ligand alterations on different levels [111].

Altogether, transition metals have various roles in cells, which are the following:

- cofactors and structural part of enzymes [112],
- maintenance of protein conformations [113],
- mediation enzyme activation/inhibition [114],
- direct participation in redox homeostasis,
- second messengers [115].

The state of the cellular metal pool is important [111]. But the identification of TMs’ targets of signalling is even more crucial [116]. However, both target prediction and identification are difficult, since the diversity and complexity of the metal sites is high [117]. To get the broader understanding of transition metal signalling see a short review: Searching for harmony in transition-metal signalling [111].

TMs have proven relation to autophagy, a metabolic program closely related to pyroptosis and ferroptosis. In various studies, autophagy incidence grew after the cells were treated with heavy metals mostly found in the group of TMs [118]. And thus, this paper provides short biochemical highlights of TMs, concerning the facts connecting TMs to ferroptosis and pyroptosis.

## Iron

Iron is an essential element for life [119], occurring mainly in its bound forms. Heme complexes, ironcontaining tetrapyrroles, are the most abundant form of iron in the human body [120]. Since heme is toxic in excess, it is bound to proteins called heme proteins, which also form heme enzymes. These facilitate gas transportation, oxidative and xenobiotic metabolism [120]. Iron-sulphur clusters (Fe-S) function as essential parts of the respiratory chain (in mitochondria) and photosynthesis (in plastids) [121]. Iron bound to proteins in non-heme form represents the main transport and storage form of iron, which will be discussed further. Iron chelators are iron-binding compounds, which affect the availability of iron. They may decrease iron uptake when present in food (for example, phytate and polyphenols) [122], and are used to treat iron-overload syndromes [123]. Recently, iron chelators are emerging as a treatment of diseases associated with oxidative stress, including atherosclerosis, cardiovascular and neurodegenerative diseases, and cancer [124]. Free state iron occurs in Fe^2+^ and Fe^3+^ forms. Balance between these two oxidative states plays a key role in maintaining of the redox balance in cells, which requires strict free iron pool regulation [125].

The uptake of dietary iron happens in the small intestine [126]. Heme bound iron entry is still not well understood [120]. Two potential mechanisms have been suggested, either a receptor-mediated endocytosis and membrane transport via a transporter [127], or a transporter mediated entry via HRG1 (heme responsive gene). This transporter was found on erythrophagosomal membranes of macrophages during iron scavenging from red blood cells [128]. However, enterocytes of the human small intestine also express HRG1, where it could serve as a heme importer from endocytic compartments [129]. Heme oxygenase (HO) then splits the porphyrin ring of the absorbed heme resulting in the release of free iron ions [130].

Free iron ions from the small intestine are absorbed by DMT1 (divalent metal transporter 1) in the Fe^2+^ form [131]. However, most dietary non-heme iron is in the Fe^3+^ form. The reduction of Fe^3+^ to Fe^2+^ happens by dietary antioxidants (i.e., ascorbate) [122] and by luminal ferrireductases such as cytochrome B (CYBRD1, also known as DCYTB) [132]. Upon cellular entry, iron binds to ferritin. Storage of iron within the cell is mediated by ferritin. It is composed of ferritin light (FTL) and heavy chain (FTH1) [133].

Efflux of iron ions is allowed by ferroportin (also known as FPN-1, IREG1, MTP1 and Slc40a1 [solute carrier family 4, member 1]), the only known exporter of iron from cells [134]. After efflux, iron is oxidised (Fe^2+^ → Fe^3+^) by hephaestin or ceruloplasmin in order to be loaded onto transferrin [135]. Transport of iron within the body is facilitated by transferrin. Transferrin bound iron is non-reactive/non-toxic unlike free iron irons. Cells salvage transferrin molecules by TFR1 (transferrin receptor 1) via receptor-mediated endocytosis [136].

**Fig. 3:**
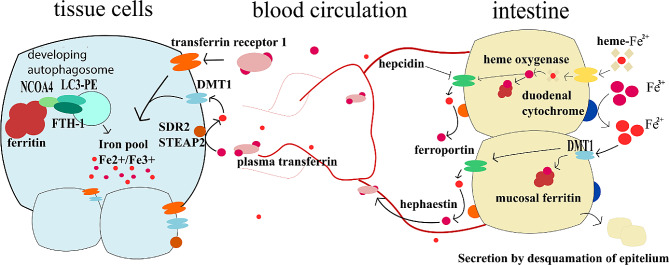
Iron metabolism. This figure summarises the main events in the iron metabolism. In small intestine lumen, iron is found in heme or ionic form. Heme-bound-Fe^2+^ enters the enterocyte via a heme transporter and is exposed to heme oxygenase, which frees the iron from heme and oxidises it to the Fe^3+^ form. Free Fe^3+^ in the intestinal lumen is reduced to Fe^2+^ thanks to the duodenal cytochrome B or dietary antioxidants. Fe^2+^ can enter the enterocyte through DMT1 (Divalent metal transporter 1). In enterocytes, iron can be stored in mucosal ferritin, or it can be effluxed into blood through ferroportin which is regulated by hepcidin. Once effluxed, Fe^2+^ is oxidised to Fe^3+^ by hephaestin or ceruloplasmin and is transported bound to plasma transferrin. Very little amounts of iron are transported in the free form. On the site of tissue cells, plasma transferrin binds to transferrin receptor 1 and enters the cells. Tissues can also uptake Fe^2+^ iron through DMT1. However, Fe^3+^ from the blood first needs to be converted to Fe^2+^ by SDR2 or STEAP2 on cells membranes

Further explanation of the iron metabolism and its regulations is out of the scope of this review, but we can recommend reading Review on iron and its importance for human health by Abbaspour N. et al. [137]. General overview of iron metabolism is drawn in the Fig. 3.

Following the discovery that O_2_ was a common cellular metabolite, it was quickly recognized that the complex of Haber – Weiss reaction could lead to generations of toxic radicals called reactive oxygen species (ROS) [138]. However, thanks to the research, we now understand that ROS are also essential for physiological cell function and signalling [138].

Haber - Weiss reaction:

$$\:{Fe}^{2+}+{H}_{2}{0}_{2}\to\:{Fe}^{3+}+{OH}^{-}+O{H}^{\bullet\:}$$

$$\:{Fe}^{3+}+{O}_{2}^{\bullet\:-}\to\:+{Fe}^{2+}+{O}_{2}$$

$$\:{O}_{2}^{\bullet\:-}+{H}_{2}{0}_{2}\to\:\:{O}_{2}+\:{OH}^{-}+O{H}^{\bullet\:}$$

Fenton reaction, although only one part of the Haber - Weiss reaction, is probably the most important reaction of the ROS metabolism. It demonstrates that Fe^2+^ has a more potent impact than Fe^3+^ [139].

Fenton reaction:

$$\:{Fe}^{2+}+{H}_{2}{0}_{2}\to\:{Fe}^{3+}+{OH}^{-}+O{H}^{\bullet\:}$$

Iron is a key player in ferroptosis. However, it also plays a significant role in pyroptosis. This connection is also highlighted by the fact that macrophages play a crucial role in iron metabolism [140]. Therefore, the role of iron in pyroptosis is definitively a field with a significant research potential.

### Iron in ferroptosis

There are several assets connecting iron to ferroptosis, present on multiple levels of the process. Firstly, ROS production is mediated by iron via two main pathways. Either directly via Fenton reaction, or indirectly as a cofactor of lipoxygenase (LOX), enabling the enzyme oxidation of PUFAs [15, 141, 142]. Especially, ACSL4 (Long-chain-fatty-acid—CoA ligase 4) and the lipoxygenases 15/15B were found to be pivotal for ferroptosis induced by iron and PUFA dyshomeostatis in dopaminergic neurons [10, 143]. Moreover, Glutathione (GSH), a cysteine containing tripeptide, helps to regulate cellular iron levels, iron transport, biosynthesis of iron-binding cofactors, and the formation of iron-GSH complexes [144]. GSH is needed in regeneration of the GPX4 pathway. Lower levels of glutathione and therefore lower activity of GPX4 leads to higher ROS levels and incidence of ferroptosis [145]. This is supported by the linkage between impaired expression of cystine/glutamate antiporter, which is essential for GPX4 regeneration, and the resulting iron dyshomeostasis manifested as an increase of ferritin and lipid peroxidation in cerebrospinal fluid. These results also indicate the involvement of ferroptosis in the development of Alzheimer’s disease [146–148].

Also, heme oxygenase 1 (HO-1) is an important ferroptosis modulating enzyme [149, 150]. In hepatocytes, excessive HO-1 expression leads to an iron overload and ferroptosis and interestingly, also to fibroblast growth factor 21 (FGF21) expression [148]. Notably, FGF21 inhibits HO-1 and thus acts as a ferroptosis suppressor [148]. HO-1 was also found to modulate ferroptosis in macrophages by regulating iron and ROS levels in response to *Bacillus Calmette-Guerin* infection [151].

p53 phosphorylation also occurs in iron overload. In the study by Zhang, P. et al., this process engaged ferroptosis, which promoted the occurrence of apoptosis [152]. Interestingly, in this study the elevation of p53 phosphorylation did not correspond to MAPK signalling pathway activation [152]. On the complex role of p53 in ferroptosis, we recommend a review by Yanging Liu and his colleagues [153].

Ferritinophagy, the autophagic turnover of ferritin, is mediated by a selective cargo receptor Nuclear receptor coactivator 4 (NCOA4) [154]. NCOA4 binds to phosphatidyl-ethanolamine conjugated light chain 3 microtubule associated protein 1 (MAP1-LC3-PE) and to FTH1 both found on the developing autophagosome membrane. This leads to the exposition of ferritin into autophagosomes [155]. Knockdown of NCOA4 and autophagy related proteins (ATGs) supresses ferritin degradation, iron accumulation and lipid peroxidation that would otherwise result in ferroptosis [156, 157]. This is further supported by the study where overexpression of mitochondrial ferritin inhibited erastininduced ferroptosis [157]. Based on this evidence, it seems that NCOA4 is related to Xc- system [158]. Belleli and his team stated that these findings provide genetic evidence of ferroptosis being a process of selective autophagic cell death [159]. This is further supported by the possibility to induce ferroptosis by ferritinophagy in multiple cancer cell lines [158]. On the topic of autophagy and ferroptosis, we recommend a review: Ferritinophagy, a form of autophagic ferroptosis: New insights into cancer treatment [160].

### Iron in pyroptosis

Iron relates to pyroptosis via ROS generation [44]. ROS may function as cellular signalling molecules, which also may alter pyroptosis. In this matter, two pathways have been identified: ROS-Tom20-Bax-Caspase3-GSDME (Tom20 – translocase of the outer membrane) [45] and ROS-NLRP3-ASC-Caspase-1 (ASC - Apoptosis-associated speck-like protein containing a CARD) [161].

In melanoma, cell iron generated ROS can induce pyroptosis [45]. ROS causes oxidation and oligomerization of the mitochondrial outer membrane protein Tom20, which recruits Bax to mitochondria. Bax then facilitates cytochrome c release into cytosol activating Caspase-3. Caspase-3 subsequently induces gasdermine E (GSDME) cleavage, which triggers pyroptosis. Since melanoma cells express a high level of GSDME, the iron-ROS-Tom20-Bax-Caspase-GSDME pathway could be a potential target for melanoma therapy [45].

Iron as a source of ROS can induce the NLRP3 inflammasome [161]. The inflammasome subsequently activates ASC and Caspase-1 leading to pyroptosis. A study suggests that neferine, a bisbenzylisoquinoline alkaloid found in lotus (*Nelumbo nucifera*), reduces ROS. Treatment with this alkaloid in endothelial cells inhibited pyroptosis induced by lipopolysaccharide-adenosine triphosphate (LPS-ATP) [161].

## Zinc

Zinc is the second most abundant TM after iron [162]. Physiologically, zinc is mostly bound to proteins and small chelators [163]. The remaining free Zn^2+^ pool is strictly controlled and always maintained. Transport of zinc into the cytosol is mediated by 14 ZIPs (Zrt- and Irt-like proteins) [164]. Export is enabled through 10 ZnT (zinc transporter) proteins [165]. Roles of zinc in biology are abundant. The most prominent, in terms of cell death modalities, are described below.

**Fig. 4:**
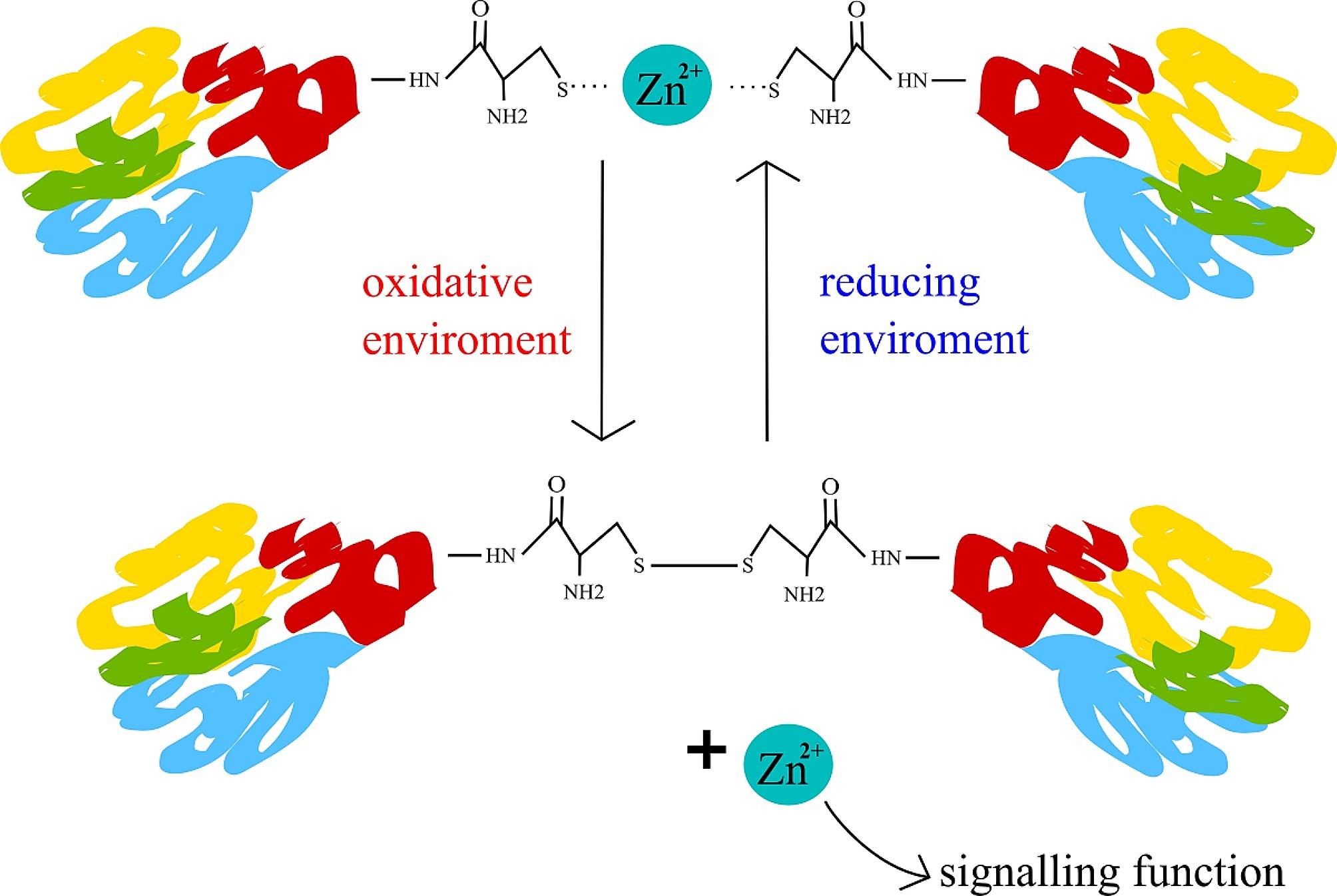
Zinc sulphur complexes. In a reducing environment, Zn^2+ ^forms complexes with the thiolate groups of cysteine. When this complex is oxidised, cysteines form a disulphuric bond, resulting in the elimination of the coordinate bond and release of Zn^2+^. Once released into the cytoplasm, Zn^2+^can also act as a second messenger

Zinc present in domains of metalloproteins enables interactions with DNA, RNA, other proteins or lipids. Furthermore, zinc can also bind subunits to control the assembly of quaternary (same protein) or quinary (different proteins) structure [166]. Metallothioneins (MT), a protein family of zinc metalloproteins, allow the control of zinc pool. MT’s zinc binding sites differ in affinities ranging over two orders of magnitude [167] enabling a delicate zinc buffering capacity [168]. In context of oxidative stress, MT can also react with superoxide and hydroxyl radicals and therefore could provide protection against oxidative stress [169]. Zinc has a unique role in redox balance as well. In vivo, zinc is only present in the Zn^2+^ state and therefore cannot directly impact redox balance [170]. However, zinc can form a coordinate bond with the thiolate group of cysteine. This zinc thiolate coordination environments are redox-active^166^, hence the sulphur in it can be oxidized [171]. This fact links zinc to redox and free radical biology, because the number of free thiol groups depends on the oxidative stress [171]. This mechanism also allows zinc to be released as a second messenger [172–174]. Such behaviour is shown in the Fig. 4.

Zinc is regarded as an antioxidant [164]. However, the term “antioxidant” is misleading since it defines a reagent with a direct substrate reduction ability and zinc does not fit this definition exactly. In biological systems, both the lack and the excess of zinc causes a pro-oxidant state, meaning that both addition and removal of zinc could balance the overall pool. The newly established balance overcomes the pro-oxidant state. Simplistically, zinc cancels pro-oxidant state, like an antioxidant. To differentiate between zinc and true antioxidants, a new term for zinc was proposed: pro-antioxidant [175].

Redox paradox is a term coined to describe the fact that dietary zinc has different effects than cellular zinc [175]. Excessive zinc in diet lowers copper uptake, therefore handicapping Cu/Zn SOD (superoxide dismutase), an important antioxidative enzyme [175]. In conclusion, excessive dietary zinc has a prooxidant effect [175]. Interestingly, zinc is also a selenium antagonist [176]. Selenium also exhibits an anticarcinogenic effect by exhibiting antioxidant properties [176]. Therefore, excessive amounts of zinc abolish the anticarcinogenic effect of selenium [177].

In the development of both ferroptosis and pyroptosis, zinc may enhance or supress these processes. This phenomenon could only be explained by the complex nature of this element in biology.

### Zinc in ferroptosis

The paradoxical nature of zinc regarding redox processes also applies in its relation to ferroptosis. Zinc seems to allow ferroptosis through ZIP7 and may contribute to both ROS generation and ferroptosis through oxidative enzymes [178]. On the other hand, zinc promotes the degradation of oxidative stress mediators [179].

In a study by Chen, P. et al., ZIP7 was found as a novel determinant of ferroptosis in breast and renal cancer cells [178]. ZIP7 controls zinc transport from the endoplasmic reticulum (ER) to the cytosol. Both genetic and chemical inhibition of ZIP7 have been shown to protect against ferroptosis [178]. This is further supported by the fact that addition of zinc promotes ferroptosis, while the zinc removal by a chelator supresses it [178]. There is a discussion about zinc affecting phospholipase A2 [180], lipoxygenase [181] or xanthine oxidase [182]. These enzymes could possibly contribute to ROS generation and the incidence of ferroptosis.

During spinal cord injury, zinc in neurons triggers the degradation of oxidative stress products through the NRF2/HO-1 and GPX4 signalling pathways. GPX4 lowers the levels of lipid peroxides, malondialdehyde (MDA) and ROS. Therefore, it inhibits ferroptosis [179].

### Zinc in pyroptosis

The ambivalent behaviour of zinc applies to pyroptosis as well. Oral zinc supplementation during infection supports the NLRP3-ASC-Caspase-1 pyroptotic axis [183], while zinc gluconate exposure also seems to upregulate NLRP3 and pyroptosis [11]. However, Zinc finger E-Box binding homeobox 2 (ZEB2) seems to alleviate pyroptosis, while Canonical transient receptor potential-6 (TRPC6) maintains zinc influx and blocks the NLRP3-ASC-Caspase-1 pyroptotic axis [184].

Oral zinc supplementation in young pigeons challenged with *Salmonella enterica serovar Typhimurium* reduces bacterial load in the liver, improves NLRP3 protein expression, and tends to increase Caspase-1 protein abundance in the jejunum [183]. Oral zinc supplementation also enhances the immune response, as well as activates Caspase-1 dependent cell pyroptosis pathways [183]. Similarly, zinc gluconate exposure results in an increase in the protein levels of NLRP3 and IL-1β in rodent olfactory neuron cell line Odora. This indicates that zinc exposure may lead to pyroptosis [11].

ZEB2 is a regulator of astrogliosis following ischemia or reperfusion injury [184]. ZEB2 promotes neuronal proliferation and regeneration by decreasing pyroptosis [184]. This finding was also verified on a rodent model, where overexpression of ZEB2 promoted astrogliosis, resulted in decreased infarct volume and improved recovery of neurological function by alleviating pyroptosis [184]. In other study by Shen, B. et al., the authors investigated the role of TRPC6 (Transient receptor potential cation channel) in pyroptosis of renal tubular epithelial cells [47]. The results show that I/R injury causes downregulation of TRPC6 both in vivo and in vitro [47]. In the OGD/R (Oxygen-Glucose Deprivation and Reoxygenation) cell model, the inhibitor of TRPC6 (SAR7334) reduced zinc ion influx, aggravated cell death, and upregulated pyroptosis-related proteins. TRPC6 inhibition exacerbated tissue damage and upregulated NLRP3, ASC, Caspase-1, IL-18, and IL-1β in the I/R injury in murine model, which could be alleviated by the administration of ZnCl_2_ [47]. The pyroptosis phenotype could be alleviated by ZnCl_2_ and intensified by zinc ion chelator [47]. Overexpression of A20 reduced the expression of pyroptosisrelated proteins, while A20 deficiency impaired the protective effect of zinc ion [47].

## Selenium

Human body converts selenium into various compounds like methylselenol or selenocysteine (Sec) [185]. Selenoproteins play an important role in antioxidant defence, formation of thyroid hormones, DNA synthesis, fertility and reproduction [185]. Hence, selenium is an essential element. Even though Se is a nonmetal element, it fulfils a special role in relationship with both ferroptosis and pyroptosis. While transition metals tend to induce necrotic cell death modalities, selenium has a protective effect (see further). In ferroptosis, selenium plays its role as a part of GPX4 [186]. In pyroptosis, lower selenium levels lead to a higher NLRP3 inflammasome activity [187]. However, the exact mechanism of this interaction is still unclear.

For more detailed information on selenoproteins biochemistry, especially their synthesis, transcription and evolutionary context, we recommend a review by Marcus Conrad and Bettina Proneth [188].

### Selenium in ferroptosis

Selenium has a protective effect and acts against ferroptosis on multiple levels. GPX4 is one of 25 selenoproteins in human body. It directly regulates ferroptosis in a suppressive manner [189, 190]. Sec presence instead of Cys in GPX4 is not essential for mice embryogenesis. However, it is essential for neuronal differentiation. This selective mechanism is based on the suppression of peroxideinduced ferroptosis [188]. Different tissues sensitivity to ferroptosis could be explained by the respective free pool of selenium. Higher pool of selenium increases the function of GPX4 and lowers the sensitivity to ferroptosis [191]. The proposed selenium-GPX4-ferroptosis axis has a central role in homeostasis of follicular helper T-cells [192]. Therefore, supplementary selenium can enhance the reaction of body to infections [192]. Fradejas, N. et al. found that isopentenyltransferase 1 (TRIT1) can isopetenylate SectRNA [193]. This interference could be new potential approach to downregulate GPX4 expression. This was supported and observed in study with FIN56 (specific ferroptosis inducer) treatment [194].

Ferroptosis stimulates transcription of protective selenoproteins [191]. Especially Sp1 (specificity protein) DNA binding is significantly induced by ferroptosis related oxidative stress [195]. However, this response seems inadequate due to the relative lack of selenium in high demand conditions [191].

High selenium levels prevent ferroptosis and ferroptosis-independent cell death modalities via the increased transcriptional response of TFAP2c (Transcription factor AP-2 gamma) and Sp1 [191]. Both indirect and direct delivery of selenium to cerebral ventricle improved the functional recovery after stroke by inhibiting ferroptosis via the mentioned axis [191]. Tat-SelPep (another Sec containing peptide) also improved outcomes after haemorrhagic stroke in mice [191, 196].

Selenium possibly modulates the Nrf2 (Nuclear factor erythroid 2-related factor 2)/GPX4 signalling pathway resulting in ferroptosis inhibition. The effect was tested on BTBR murine model (Autism spectrum disorders model, BTBR T^+^*Itpr3*^*tf*^*/J*), where it also exhibited a beneficial effect on autismrelevant behaviour [197].

Ebselen is an organoselenium compound and serves as a pharmacological GPX mimetic [196]. It exhibits a hydroperoxide-reducing activity like GPX. In vivo experiment confirmed that ebselen stays intact and does not provide selenium for GPX4 [196]. Ebselen itself was found to be able to prevent ferroptosis in neurons [198].

### Selenium in pyroptosis

Selenium protects against pyroptosis as a part of GPX4 [199], by altering the PI3K/AKT/PTEN axis [200], by inhibiting the NLRP3 inflammasome, and of course through its antioxidant properties [201]. Selenium is utilised by GPX4, which attenuates cadmium-induced ferroptosis and pyroptosis in sheep kidney [200]. Selenium also antagonises Cd-induced pyroptosis, autophagy and apoptosis by altering the PI3K/AKT/PTEN signalling in heart [200]. Yeast selenium, one of the most effective organic selenium compounds [202], exhibits an antioxidant effect on chicken liver, counteracting the cadmium induced pyroptosis [203]. A selenium-modified phytosomal tripterine was found to reduce cytotoxicity and inflammation by inhibiting the NLRP3 inflammasome and pyroptosis [201].

On the other hand, Se deficiency itself can lead to pyroptosis via ROS-NLRP3-IL-1β axis [204]. Also, during Se depletion, both TXNRD3 (Thioredoxin Reductase 3) [205] and miR-1656 target NLRP3 activation and pyroptosis [48]. In pig spleen, selenium deficiency causes pyroptosis via ROS/NLRP3/IL-1β signalling pathway [204]. A selenoprotein thioredoxin reductase 3 (TXNRD3) was found to be associated with pyroptosis and necrosis. In TXNRD3 knock-out mice the expression of NLRP3, Caspase-1, RIPK3 (Receptor-interacting serine/threonine-protein kinase 1), and MLKL (Mixed Lineage Kinase Domain-Like Pseudokinase) increased significantly [205]. However, overexpression of TXNRD3 leads to calcium outflow-induced oxidative stress followed by necrosis and pyroptosis [205]. A micro-RNA miR-1656 targets GPX4 leading to NLRP3 activation and pyroptosis in selenium deficient broiler kidney tissues [48].

## Copper

In vivo, copper exists in two stable ion forms (Cu^+^ and Cu^2+^), making redox reactions possible [206]. Therefore, the maintenance of copper homeostasis is crucial [207]. Cellular influx of copper is mediated via the Cu transporter 1 (CTR1) [208, 209]. While Cu-ATPases (ATP7A and ATP7B) facilitate efflux of copper [210]. Intracellular copper is either chelated by metallothionein, as storage, or bound to Cu chaperones [211]. These chaperones also serve as transporters - taking copper ions to their targets [210]. Many oxido-reductases require copper to function properly. The notable ones are Cu/ZnSOD, ceruloplasmin, cytochrome c oxidase, lysyl oxidase, tyrosinase and dopaminebetahydroxylase [212]. Copper also impacts iron homeostasis via ferroxidases haephaestin and ceruloplasmin [213]. These copper-rich enzymes, each containing 6 atoms of copper, are essential for electron transfer allowing ferroxidase activity [214].

Copper is proven to be related to both ferroptosis and pyroptosis. In the case of copper in ferroptosis, there is a common mechanism found in most TMs – the disruption of iron homeostasis. However, in this case the redox activity of copper itself should not be overlooked.

### Copper in ferroptosis and pyroptosis

Copper induces the autophagic degradation of GPX4 resulting in ferroptosis [215]. On the other hand, copper depletion enhances mitochondrial perturbation. This process depletes the antioxidative mechanism leading to ferroptosis as well [216]. Cuprizone is a synthetic copper chelator. In experimental animals, it is used to cause demyelination [217]. Jhelum P. et al. proved that cuprizone diet-induced destruction of oligodendrocytes is caused by ferroptosis [217]. They showed that chelating copper leads to the expression of molecules rapidly mobilizing iron from ferritin. The released iron then shall trigger iron-mediated lipid peroxidation resulting in ferroptosis [217]. To conclude, it seems both copper overload and depletion lead to ferroptosis. This highlights the importance of copper homeostasis.

Copper induces pyroptosis in pig jejunal epithelial cells. Copper excess causes endoplasmic reticulum stress via IRE1α-XBP1 (Inositol-requiring enzyme 1, X-box binding protein spliced) pathway and this pathway subsequently mediates pyroptosis [49].

Recently, Peter Tsvetkov and his team distinguished a new copper mediated cell death modality cuproptosis [49, 218]. In this case, copper binds directly to lipoylated components of the tricarboxylic acid cycle causing their aggregation. The subsequent loss of iron-sulphur cluster proteins leads to proteotoxic stress and cell death [49, 218].

## Molybdenum

Molybdenum (Mo) is a vital micronutrient that acts as an enzyme cofactor. In humans, 4 enzymes are known to contain Mo: xanthine oxidase, sulphite oxidase, aldehyde oxidase and mitochondrial amidoxime-reducing component (mARC) [219, 220]. Once in body, Mo primarily accumulates in the liver and kidneys. The kidneys are particularly prone to Mo toxicity, as approximately 50% of this element is metabolised there. Excessive Mo intake has been linked to chronic renal failure [221], poor growth, anaemia, diarrhoea, significant functional and morphological damage in several organs, and even death [222].

Mo compounds can catalyse ROS generation [219]. This fact links them to ferroptosis, but also to possible cancer treatment [219]. However, the role of this TM is unclear in pyroptosis. Up to date published results were obtained in co-induction with cadmium (Cd) [50, 223, 224]. Therefore, the role of Mo in these cases, as well as the interaction with Cd, is unclear.

### Molybdenum in ferroptosis

MoO_4_^2−^ as a catalyst can turn hydrogen peroxide H_2_O_2_ (a common ROS in malignant tumours) to singlet oxygen (^1^O_2_). However, as authors of this recent study state, molybdate ions are not suitable for redox-based cancer treatments [219]. Their study presents the molybdenum sulphide (MoB) nanocatalysts. MoB simultaneously generates ^1^O_2_ and superoxide anions (O_2_•^−^) from H_2_O_2_. Interestingly, MoB-created ROS induce lipid peroxidation and result in ferroptosis. Hence MoB could present a possible efficient cancer therapy [219].

### Molybdenum in pyroptosis

Molybdenum overexposure can lead to oxidative stress and pyroptosis [50]. Moreover, Mo induces autophagy in the kidneys as well [225]. Mo and Cd co-induce pyroptosis in duck brains. The mechanism involves the inhibition of the NRF2-mediated antioxidant defence response [223]. Other possible mechanism is PTEN/PI3K/AKT axis found in Mo and Cd co-induced pyroptosis and apoptosis in the livers of Shaoxing ducks (*Anas platyrhynchos*) [224].

## Cobalt

Although cobalt is an essential element as the metal core of vitamin B12, excessive cobalt (Co) exposure has various adverse health effects [226]. Cobalt occurs in alloys, batteries and also in artificial joints, from where it can be up-taken by a human body [226, 227].

Cobalt induces ferroptosis via a Fenton-like reaction [228] and pyroptosis via ROS-NLRP3-Caspase-1 pathway [229]. The same effect is induced by cobalt nanoparticles, which we mentioned here due to their clinical importance [227]. These nanoparticles are the result of the wear of artificial joints [227]. However, other TM nanoparticles are out of scope of our review.

### Cobalt in ferroptosis

Co ions in water produce ROS in a Fenton-like reaction. Free Co^+^ ions can generate $\:\text{O}{\text{H}}^{{\bullet\:}}\:$radicals [228].

$$\:{Co}^{+}+{H}_{2}{0}_{2}\to\:{Co}^{2+}+{OH}^{-}+O{H}^{\bullet\:}$$

On the other hand, the reaction of free Co^2+^ with H_2_O_2_ does not generate any significant amount of $\:\text{O}{\text{H}}^{{\bullet\:}}$ radicals. However, biological chelators such as GSH or beta-ananyl-3-methyl-L-histidine alter the oxidation-reduction potential of Co^2+^ enabling another Fenton like reaction [228].

$$\:{Co}^{2+}\left(chelated\right)+{H}_{2}{0}_{2}\to\:{Co}^{3+}+{OH}^{-}+O{H}^{\bullet\:}$$

Supporting these claims, Co particles from endoprostheses increase ROS, Fe^2+^ levels, lipid peroxidation, GSH consumption and inhibit GPX4 activity [227]. These processes induce a ferroptosis-like cell death. Importantly, α-lipoic acid (ALA), a natural antioxidant, a scavenger of free radicals and a chelator of toxic metals, can efficiently counteract the effects of Co particles [227].

### Cobalt in pyroptosis

Wear particles from implant surfaces induce periprosthetic osteolysis. A study by Xue S. et al., focuses on cobalt-chromium-molybdenum implant wear particles (CoPs) [229]. They found that CoPs exposure and accumulation in macrophages induces ROS generation and subsequently NLRP3dependent pyroptosis [229]. This, in turn, also stimulates the release of pro-inflammatory cytokines such as IL-18, IL-1β, and HMGB1 (High-mobility group box 1). CoPs also led to mitochondrial damage in macrophages, accelerating ROS production and NLRP3-dependent pyroptosis [229].

Cobalt can induce acute kidney injury (AKI) through hypoxia-reoxygenation injury (HRI) [230]. Liu, W. et al., induced HRI-AKI by CoCl_2_ both in vitro and in vivo [230]. According to their study, GSDMEmediated pyroptosis was involved in cell damage under HRI-AKI. GSDME is cleaved by Caspase-3/8/9 leading to pyroptosis [230]. Autophagy also occurs in HRI-AKI. In this case autophagy also induces a GSDME-mediated pyroptosis via apoptotic pathways [230].

## Nickel

Nickel (Ni) has a dual nature of an essential as well as a toxic element [231]. In higher organisms, a higher perinatal mortality, changes in grooming behaviour, and a decreased growth are also present in the absence of Ni [231]. Nickel depletion, although rare, reduces intra-uterine development and iron adsorption leading to anaemia in humans [231]. Ni is associated with allergies, carcinogenesis and infectious agents, which rely on nickel-based enzymes [232]. However, there is little evidence of Ni accumulating in the food chain. Moreover, Ni, unlike Pb, is not a cumulative toxin for humans. Almost all cases of acute poisoning are due to nickel carbonyl [233].

### Nickel in ferroptosis

In mice, Ni causes histopathological alterations of liver and an increase in AST (aspartate aminotransferase) and ALT (alanine aminotransferase) serum levels [234]. There is also an increase in MDA (Malondialdehyde) production, while total antioxidant capacity and GSH content are reduced [234]. Then ROS levels increase, and mitochondrial membrane depolarizes. Meanwhile, Ni increases iron content, upregulates cyclooxygenase 2 and down-regulates GPX4, FTH1 and NCOA4 resulting in ferroptosis [234].

### Nickel in pyroptosis

Selenoprotein M (SelM) is a common antioxidant protein found in various tissues [235]. In SelM knockout mice’ spleen, Ni induces the expression of ASC, AIM2, NLRP3, Caspase-1, IL-18 and IL-1β and results in pyroptosis [236]. Interestingly, in the same experiment, melanin was found to alleviate the impact of Ni exposure and offered protection against pyroptosis [236].

## Platinum

Elemental Platinum is very biologically inert and therefore of a relatively small interest as a part of a metabolism. However, its compounds are used as common chemotherapeutics and exhibit a variety of interesting properties. This is also the field, where the connection between Pt and ferroptosis and pyroptosis is established. A well-written platinum (Pt) biochemistry review regarding Pt coordination complexes and Pt-DNA interaction was published by Douple and Richmond [237]. Generally, the toxicity of noble metals such as Pt is still unclear [238].

### Platinum in ferroptosis

Cis-platin administration induces ferroptosis in multiple ways. Cis-platin was found to inactivate GPX and deplete GSH in A549 and HCT116 cells resulting in ferroptosis [239]. Furthermore, cis-platin increases the expression of both transferrin receptor and ferritin, which results in increased cellular iron levels [240]. Cis-platin treatment was followed by an increased expression of ferroptosis markers, COX2 (cyclooxygenase-2) and 4hydroxynonenal (4-HNE) and ferroptosis respectively [240]. Ferrostatin-1 or VPA (valproic acid) decreases the occurrence of ferroptotic phenotype after cis-platin administration [240, 241]. Thus, cisplatin treatment together with ferroptosis induction makes a promising strategy for ovarian cancer [242].

Interestingly, the activation of vitamin D receptor seems to have a protective effect in cis-platin induced ferroptosis [243]. Please note, that cis-platin also induces other cell death modalities, i.e., apoptosis and necrosis [244].

Colorectal cancer cells are often resistant to oxaliplatin treatment, a cis-platin derived drug [245]. This resistance can be reversed by ferroptosis induction, possibly by the mechanisms of lipid peroxidation, and iron metabolism disruption [246]. Oxaliplatin sensitivity depends on the KIF20A-NUAK1 (Kinesin family member 20 A, SNF1-like kinase 1) and GSK3β-NRF2 (glycogen synthase kinase 3β, nuclear factor erythroid 2-related factor 2) signalling pathways in colorectal cancer cells [247].

Novel cis-platin derived drugs are being synthetized. Their properties are often targeted to evade side effects or to overcome resistance in cancer cells. For example, platinum(IV) complexes conjugated with ligustrazine-based chalcones showed promising anticancer effects by inducing apoptosis and ferroptosis through accumulation of LPOs and inhibiting xCT-GPX4 axial pathway [248]. Platin B is another drug derived from cis-platin by addition of 4-carboxylphenylboronic acid [249]. This acid is a powerful GSH scavenger [249]. Therefore, the administration of Platin B leads to cellular GSH depletion, which triggers ferroptosis [249].

### Platinum in pyroptosis

Cis-platin can induce pyroptosis in tumour cell in many possible mechanisms. In triple negative breast cancer cells, cis-platin upregulates the long non-coding RNA maternally expressed gene 3 (MEG3) [51]. This subsequently activates the MEG3-NLRP3-Caspase-1-GSDMD pyroptotic axis [51]. Other study confirms that the cleavage GSDMD after cis-platin administration induces acute kidney injury by pyroptosis [52].

Furthermore, in oesophageal cancer cells cis-platin targets calpain-1 (CAPN1) and calpain-2 (CAPN2) [53]. In this particular study, high expression of CAPN-1 and CAPN-2 was associated with favourable clinical outcomes after cis-platin treatment [53]. In this particular case, scientists discovered that cis-platin induces the CAPN1/CAPN2-BAK/BAX-Caspase-9-Caspase-3-GSDME pathway [53]. Caspase-3GDSME activation was also described in another chemotherapeutic agent paclitaxel [54]. However, cis-platin seems to be a stronger inductor of pyroptosis than paclitaxel [54].

Interestingly, cis-platin exhibits a protective effect by inhibiting Caspase-3-mediated GSDME-derived pyroptosis in noncancerous tissues of squamous cell carcinoma patients [250].

Lobaplatin induces pyroptosis by GSDME cleavage and Caspase-3 activation in colon cancer cells [55]. Knock-out of GSDME results in a switch from pyroptosis to apoptosis. Lobaplatin treatment increases ROS levels and JNK (c-Jun N-terminal kinase) phosphorylation, which can be reversed by ROS scavenger NAC (Nacetylcysteine) [55]. JNK activation recruits Bax to mitochondria, resulting in cytochrome c release to cytosol, followed by Caspase-3/9 cleavage and pyroptosis [55]. Lobaplatin induces Caspase-3-GSDME pyroptosis also in cervical cancer cells [56]. Lobaplatin can also induce pyroptosis by degradation of cell inhibitor of apoptosis protein-1/2 (cIAP1/2) in nasopharyngeal carcinoma cells [57]. While the recovery of cIAP1/2 inhibits pyroptosis. Unsurprisingly, the inhibition of cIAP1/2 by a specific antagonist dramatically enhanced lobaplatin induced pyroptosis [57]. However, the inhibition of ripoptosome (RIPK1/Caspase-8/FADD), of ROS and Caspase-3 cleavage cancels the before mentioned synergistic effect [57].

Targeting the cyclic GMP-AMP synthase-stimulator of the interferon gene (cGAS-STING) pathway is a potent anticancer immunotherapeutic strategy [251]. Ling, Y. et al. developed two PtII complexes (Pt1 and Pt2), which photoactivate the cGAS-STING pathway, while also inducing pyroptosis in cancer cells. This process triggers a potent anticancer immune response both in vitro and in vivo [251].

## Cadmium

Cadmium (Cd) is a non-essential toxic heavy metal [252]. Chronic exposure leads to systemic toxicity and causes various cancers (mostly in the lung, prostate, breast, pancreas, nasopharynx and kidney) [252]. Cadmium’s long biological half-life (25–30 years) leads to accumulation in the human body for decades [253]. Interestingly, a corelation was found between prostate cancer progression and rising Cd serum levels [254].

Cadmium disrupts protein stability [255]. This fact connects Cd to both ferroptosis and pyroptosis. However, this is presented in a very high variability of interactions [255]. Therefore, the specificity of these interactions seems very low. Nevertheless, cadmium still causes these cell death modalities. A better understanding of cadmium-protein interactions is needed, this fact provides a wide area for further research.

### Cadmium in ferroptosis

Cd can induce ferroptosis in multiple ways. Cd activates heme oxygenase 1 (HO-1) leading to free iron release from heme [256]. Endoplasmic reticulum stress-mediated ferritinophagy caused by Cd exposure, also raises cellular free iron [257]. Interestingly, Cd also lowers testosterone synthesis by promoting ferroptosis and preventing the fusion of autophagosome with lysosome [256, 258–262].

Cd induced ferroptosis triggers the PERKeIF2α-ATF4-CHOP (RNA-like endoplasmic reticulum kinase, Eukaryotic Initiation Factor 2 alpha, Activating Transcription Factor 4, CCAAT-enhancer-binding protein homologous protein) pathway, corresponding with the endoplasmic reticulum stress theory. It is further supported by the suppression of ferroptosis incidence after ER stress inhibition by iron chelating [46, 257].

Cd also causes lipid peroxidation and inhibits the Kelch-like ECH-associated protein 1 (KEAP1)NRF2ARE (antioxidant response element) signalling pathway [263]. This results in ferroptosis, which also impairs the behaviour and growth of the tested Drosophila [263].

Cd modulates the miR-34a-5p/Sirt1 axis. In PC12 (rat pheochromocytoma) cells Cd promotes *miR-34a-5p* to target Sirtuin 1 resulting in cytotoxicity [264]. Cytotoxicity, as well as both apoptosis and ferroptosis is attenuated by *miR-34a-5p* knock-out in Cd exposure [264].

Cd alters the Gpx4/Ager (receptor for advanced glycation endproducts)/p65 axis. In a study on pancreatic β-cells, a treatment with Cd induced ferroptosis and Ager/p65 related inhibition [265]. Interestingly, ferroptosis inhibitor Fer-1 disrupts the Ager activation and ferroptosis [265].

Vitamins A, D and E reduce lipid peroxidation, oxidative stress, and also inflammatory responses in Cd exposed MCF7 (Michigan Cancer Foundation-7, breast cancer) cells [266]. While vitamins A and E are known antioxidants, vitamin D surprisingly works in this case as well. This might indicate a connection between vitamin D, oxidative stress and maybe even ferroptosis.

### Cadmium in pyroptosis

Cd contributes to pyroptosis in different manners. Cd exposure results in a dose-dependent increase in ROS generation [58, 267]. In duck renal tubular epithelial cells, this results in a percentual increase of pyroptotic cells, lactate dehydrogenase (LDH), NO, IL-18 and IL-1β releases and relative conductivity [268]. The whole process is accompanied by the upregulation of mRNA for PTEN (phosphatase and tensin homolog), ASC, NLRP3, NEK7 (NIMA-related kinase 7), Caspase-1, GSDMA, GSDME, IL-18 and IL-1β [268]. With the corresponding increase in protein levels of PTEN, Caspase-1, p20, NLRP3, ASC, GSDMD. While PI3K, AKT and p-AKT expression levels decreased [268].

At high levels, Cd seems to increase the expression of NLRP3, Caspase-1, IL-1β, and IL-18, while this process can be attenuated by ROS inhibitor NAC [58]. This supports the fact that the NLRP3 inflammasome and Caspase-1 expression is a common response to pyroptosis in endothelial cells challenged by toxic chemicals, with ROS production playing a central role [58]. GSDME executes pyroptosis induced by Cd toxicity, ROS generation and NLRP3 activation in a triple-negative breast cancer (TNBC) MDAMB-231 cell model [58]. From this study it also seems, that low concentrations of Cd result in apoptosis, while high concentrations contribute to pyroptosis accompanied by LDH release. z-VAD, a broad-spectrum Caspase inhibitor, inhibits all the previously mentioned processes [58]. Additionally, Cd also activates the p38-MAPK signalling pathway, leading to nuclear translocation of NF-κB/p65 (Nuclear factor kappa-light-chain-enhancer of activated B cells) and the expression of proinflammatory cytokines. Interestingly, Cd caused cell cycle arrest in S phase with cyclin alterations [58]. Subsequently, the expression of pro-inflammatory cytokines is activated, and these are then also contributing to pyroptosis [59]. Cd activates IRE-1α/XBP-1 (X-box binding protein 1) branch of endoplasmic reticulum stress in human proximal tubular epithelial HK-2 cells. This induces Caspase-1 and NLRP3 inflammasome-dependent pyroptosis, therefore causing nephrotoxicity [269].

## Mercury, lead, uranium

### Mercury

Mercury is a toxic heavy metal. Human exposure mostly results from fish consumption and dental amalgams [270]. Organic mercury compounds can induce apoptosis and necrosis in a variety of cell types [271]. However, mercury (Hg^2+^) and methylmercury (CH_3_Hg^+^) exposure results in ferroptosis [271]. In human embryonic kidney 293T cells, HgCl_2_ and CH_3_HgCl trigger ferroptosis, explaining their nephrotoxicity [271]. Hg^2+^ and CH_3_Hg^+^ stress down-regulates GPX4 expression. CH_3_HgCl also directly binds to the selenol group (-SeH) of GPX4 inhibiting its activity [271]. Remarkably, selenite supplementation enhances GPX4 activity and antagonises the cytotoxicity of CH_3_HgCl [271].

In LPS treated mice, mercury disrupts mitochondrial ROS production [272]. This further leads to the inhibition of ASC pyroptosome and GDSMD cleavage, resulting in impaired inflammatory response and attenuated pyroptosis [272].

### Lead

Lead (Pb) is a toxic environmental pollutant associated with adverse effects on human health such as neurotoxicity, carcinogenicity, and others [273]. Pb enhances blood-cerebrospinal fluid barrier (BCSFB) permeability and accumulates in brain tissue, leading to its dysfunction [274]. Choroid plexus (CP) cells are the main components of the BCSFB crucial to its functions [274]. Ferroptosis was identified as the main cell death modality in primary cultured CP cells after Pb exposure [274]. Under these conditions, 16 ferroptosis-related genes show alteration of expression [274]. Notably, GPX4, Slc7a11, TFR, and Slc40a1 [274]. Moreover, inhibition of ferroptosis enhances CP cell’s viability and reduces BCSFB permeability [274].

Pb increases NLRP3 expression and Caspase-1 cleavage, and also increases autophagy and NF-κB phosphorylation [275].

### Uranium

Uranium (U), a toxic and radioactive metal, presents an environmental and health matter of concern [276]. Uranium was found to be connected to ferroptosis in two ways. U leads to iron accumulation and a decrease in GPX activity in Vicia faba roots [277]. These factors trigger ferroptosis like cell death [277]. The researchers also found that the cell deaths were promoted by addition of Fe, while inhibited by antioxidants (ferrostatin and Vitamin E) and iron chelator ciclopirox olamine [277].

Researchers investigated the effects of long-term low dose gamma radiation from U tailings on gene expressions in the AHH-1 lymphocytes cells [278]. It is important to note that gamma radiation itself can generate ROS [279]. In the experiment, the expression of *TFR*,* SLC3A2*,* SLC39A*,* FTH1*,* ACSL4*, and *GPX4* genes was found sensitive to low-dose radiation. These genes represent the ferroptotic pathway, hence it is safe to assume connection between gamma radiation and ferroptosis [278].

## Conclusions

In this review, we described the role of transition metals in ferroptosis and pyroptosis. Ferroptosis is a cellular death modality caused by the oxidative stress-induced lipid membrane disruption. The whole process occurs when oxidative stress presented by ROS is greater than the capacity of the cells’ antioxidant mechanisms. The main representants of antioxidant mechanisms, in the case of ferroptosis, are the Xc- system and the GPX4 axis. As the name ferroptosis suggests, ferrous ions are the main cause of oxidative stress, via the Fenton reaction. However, other metals than iron also interact with this pathway. These interactions are presented in 3 main ways:

- metals create ROS themselves, or lead to the creation of ROS as enzyme cofactors and by signalling pathway alterations,
- metals impair the antioxidant systems (GPX4 axis),
- metals affect iron metabolism, increase its availability for the Fenton reaction and result in the ROS formation.

Meaning, some increase the incidence of ferroptosis, while others act in a protective manner (for example Se). Nickel, mercury, lead, and uranium are a group of TMs, which cause ferroptosis by a dysregulation of iron homeostasis. Interestingly, zinc has a dual effect.

**Fig. 5:**
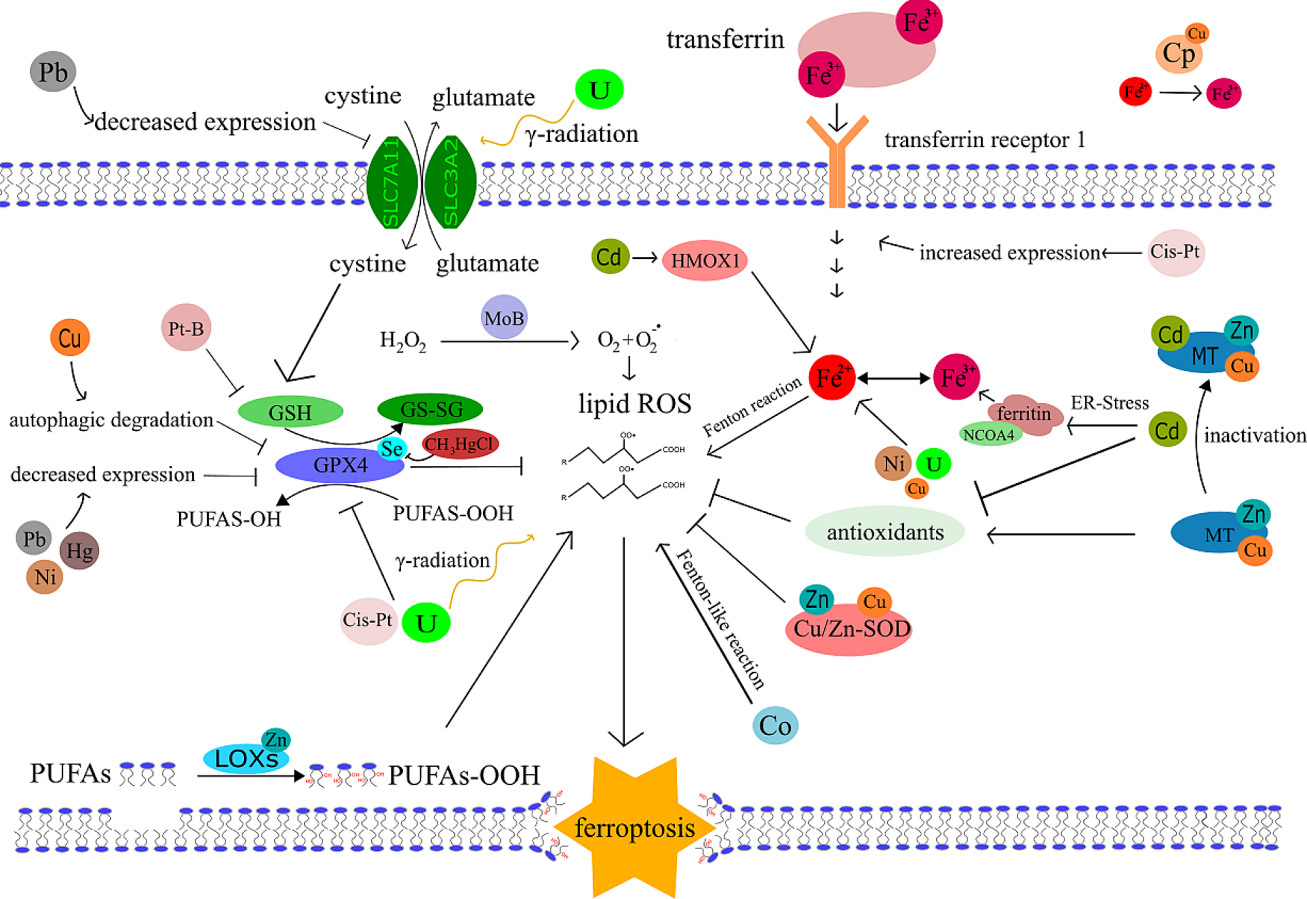
Ferroptosis and significant transition metals overview. In this figure we summarise an overview of main roles of various transition metals in ferroptosis documented in this review. Iron (Fe) generates hydroxyl radicals by undergoing the Fenton reaction. The iron pool is supplemented by NCOA4 mediated ferritinophagy or by iron import through transferrin receptor 1. Zinc (Zn) is found in abundant superoxide dismutase (Cu/Zn-SOD) and in metallothionein (MT). Cu/Zn-SOD scavenges superoxide and MT has a pro-antioxidant effect. Zinc is further present in lipoxygenase (LOX) generating PUFAS-OOH. Selenium (Se) acts as a ferroptosis suppressor mainly by the presence in GPX4. Copper (Cu) is found in Cu/Zn-SOD and MT. Ceruloplasmin (Cp) has a key role in the maintenance of Fe^2+^and Fe^3+^pool balance. Cu was also found to induce an autophagic degradation of GPX4. Molybdenum (Mo) participates in the form of molybdenum sulphate (MoB) on the catalytic generation of superoxide radical. Cobalt (Co) undergoes Fenton-like reaction generating ROS. Nickel (Ni) dysregulates iron homeostasis decreases expression of GPX4. Platinum (Pt) is represented by the platinum derived chemotherapeutics – cis-platin (Cis-Pt) and its derivate Platin B (Pt-B). Cis-platin alone increases expression of transferrin receptor 1 and inhibits GPX4. 4-carboxylphenylboronic acid added in Platin B is potent GSH scavenger which depletes GPX4 from its crucial substrate. Cadmium (Cd) activates Heme oxygenase 1 (HMOX1), inactivates MT and various antioxidants and induces ER stress mediated ferritinophagy. Mercury (Hg) decreases the expression of GPX4. CH3HgCl also directly binds to the selenol group (-SeH) of GPX4 inhibiting its activity. Lead (Pb) decreases expression of GPX4 and SLC7A11 transport system. Uranium (U) dysregulates iron homeostasis and by γ-radiation generates ROS and alters expression of SLC3A2. All the pathways are mentioned in the following summarising Table 1

To give a better perspective on each metal’s effects a graphics of the above mentioned ferroptosis pathways is presented in this work (Fig. 5). Additionally, every metal’s pathway is listed in Table 1 to complement Fig. 5 in a more detailed fashion.

In conclusion, the influence of metals on ferroptosis is complex and has a lot of implications. The understanding of these processes is crucial for developing successful strategies to treat health issues concerning inflammation, immune response pathology, carcinogenesis, and many others.

Pyroptosis is characterised by the activation of Caspase-1,4,5 or 11 resulting in gasdermin cleavage and pore formation. Originally, this cell death modality was discovered as a response to PAMPs – bacterial toxins. PAMPs can trigger pyroptosis via canonical and non-canonical pathways. After the activation of the canonical pathways (with PAMPs or DAMPs), the inflammasome is created, leading to downstream activation of Caspase-1. In the case of the non-canonical pathway, bacterial endotoxins activate Caspese-4 or -5. Both pathways end in the Caspases-mediated cleavage of GSDMD (or other gasdermins), leading to pore formation and subsequent lysis of the cell. However, both pathways may be significantly altered and even initialized by metal ions. These interactions are following:

- metals directly, or via signalling pathways interact with inflammasome formation,
- metals induce ROS which contribute to inflammasome activation,
- metals activate Caspase-3, which catalyses the non-canonical pathway.

**Fig. 6:**
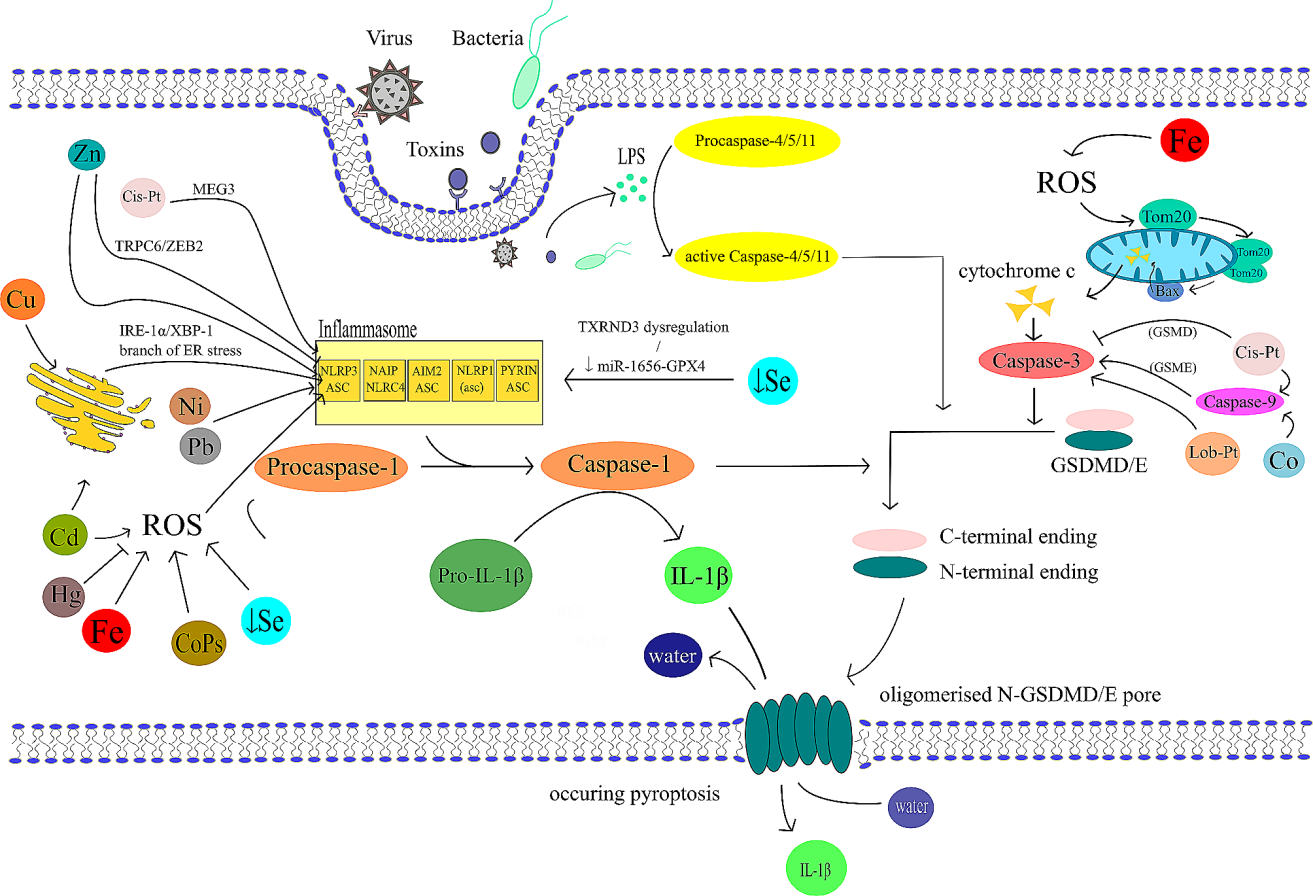
Pyroptosis and significant transition metals overview. In this figure we summarise an overview of main roles of various transition metals in pyroptosis documented in this review. Iron (Fe) generates ROS, which activates ROS-NLRP3-ASC-Caspase-1 or ROS-Tom20-Bax-Caspase-GSDME pathway. Zinc (Zn) contributes to pyroptosis by NLRP3-ASC-Caspase-1 or alleviates it by the TRPC6-NLRP3-ASC-Caspase-1 or ZEB2. Selenium (Se) depletion leads to the increase in free ROS or TXRND3 dysregulation. Se deficiency induces upregulation of miR-1656 leading to heightened expression of pyroptosis associated genes such as NLRP3 by suppressing GPX4 release. Copper (Cu) activates inflammasome by the IRE1α/XBP1(ER stress)-NLRP3 axis. Cobalt (Co) nanoparticles induce the ROS-NLRP3-ASC-Caspase-1 pathway. Cobalt also induces GDSME-Caspase-3/8/9 activation. Nickel (Ni) up-regulates ASC, AIM2, NLRP3, Caspase-1, IL-18 and IL-1β. Platinum (Pt) is represented by the most used platinum derived chemotherapeutics – cis-platin (Cis-Pt) and its derivate lobaplatin (Lob-Pt). Cis-Pt induces the CAPN1/CAPN2-BAK/BAX-Caspase-9-Caspase-3-GSDME pathway. Cis-Pt also activates the MEG3-NLRP3-Caspase-1-GSDMD axis. Cis-Pt inhibits Caspase-3-GSDMD pathway in non-cancerous cells. Lobaplatin activates Caspase-3-GSDME pathway. Cadmium (Cd) activates both ROS-NLRP3-Caspase-1 and IRE1α/XBP1(ER stress)-NLRP3 pathways. Lead (Pb) increases NLRP3 and Caspase-1 expression. Mercury (Hg) disrupts ROS production and inhibits the ASC pyroptosome and GDSMD cleavage. Lead (Pb) increases NLRP3 expression and Caspase-1. Molybdenum (Mo) role in pyroptosis is unclear and therefore we didn´t visualised its role in the scheme. Uranium (U) role in pyroptosis wasn´t yet documented. All the pathways are mentioned in the following summarising Table 1

Like in case of ferroptosis, a summarising graphics (Fig. 6) depicting the effects of metals on pyroptosis was created. All the relevant pathways are also listed in Table 1. Notably, Table 1 is no comparison between ferroptosis and pyroptosis, both cell death modalities are unique. This table (Table 1) summarises and gives a more detailed legend to Figs. 5 and 6.

To conclude, both ferroptosis and pyroptosis are important pathophysiological mechanisms. Transition metals interfere with these cell death modalities. Their homeostasis is crucial in induction both triggering and inhibiting pathways. These findings highlight the importance of TMs in cell biology. Currently, many articles are published on this topic. This promises further clarification of some other regulatory pathways, or even progress in the treatment of serious diseases caused by damage to these cell death modalities regulation together with corrupted metal homeostasis.

**Table 1:** The relation of transition metals to pyroptosis and ferroptosis. Please note that the protective factors and pathways are underlined

Transition metal	Pathways related to ferroptosis	Pathways related to pyroptosis
Fe	- ROS generation (Fenton reaction) [138, 139] - LOX cofactor [8] - GSH-GPX4 ROS suppression [145] - heme oxygenase [148, 151] - NCOA4 ferritinophagy [157] - p53 [152]	- ROS-Tom20-Bax-Caspase-GSDME [45] - ROS-NLRP3-ASC-Caspase-1 [161]
Zn	- ZIP-7 [178] - NRF2/HO-1/GPX4 defence pathway [179] - lipoxygenase [181] - MT antioxidative function [169]	- NLRP3-ASC-Caspase-1 [11, 183] - ZEB2 [184] - TRPC6-NLRP3-ASC-Caspase-1 [47]
Se	- GPX4 [188, 192] - TRIT1 isopenthenylation of GPX4 Sec-tRNA [193] - TFAP2c [191] *-*Sp1 [195] *-*NFR2 (protective) [197] - Ebselen [198]	- GPX4 PI3K/AKT/PTEN [200] - GPX4-NLRP3 inhibition [201] - selenium deficiency ROS/NLRP3/IL-1β [204] - selenium deficiency TXRND3 dysregulation [205] - selenium deficiency-miR-1656-GPX4-NLRP3 [188]
Cu	- autophagic GPX4 degradation [215] - mitochondria perturbation and loss of antioxidant capacity in copper depletion [216] - dysregulation of iron homeostasis [217]	- IRE1α/XBP1(ER stress)-NLRP3 [49]
Mo	- catalytic generation of ROS [219]	- unclear - possibly PTEN/PI3K/AKT [224] or Nrf2-mediated antioxidant defence response inhibition [223]
Co	- Fenton-like reaction [228]	- ROS-NLRP3-Casspase-1 [229] - GDSME-Caspase-3/8/9 activation [230]
Ni	- dysregulation of iron homeostasis [234] - downregulation of GPX4, FTH1, NCOA4 (Mitochondria damage and ferroptosis involved in Ni-induced hepatotoxicity in mice) [234]	- in SelM KO mice Ni up regulates ASC, AIM2, NLRP3, Caspase-1, IL-18 and IL-1β [239]
Pt	- cis-platin inactivates GPX and depletes GSH [239]. - cis-platin increases the expression of transferrin receptor and ferritin, which increases cellular iron levels [240] - activation of vitamin D receptor alleviates cis-platin induced ferroptosis [243] - cis-platin bound 4-carboxylphenylboronic acid scavenges GSH [249]	- cis-platin activates the MEG3-NLRP3-Caspase-1-GSDMD axis [51] - cis-platin induces the CAPN1/CAPN2-BAK/BAX-Caspase-9-Caspase-3-GSDME pathway [53] - Cis-platin inhibits Caspase-3-GSDMD pathway in non-cancerous cells [250] - Lobaplatin-Caspase-3-GSDME [56] - Lobaplatin-JNK-Bax-Cytochrome c-Caspase-3/9, Lobaplatin-cIAP1/2 [57] - Pt II complexes- cGAS-STING pathway [251]
Cd	- dysregulation of iron homeostasis by HO-1 activation [256] - ER stress mediated ferritinophagy [257] - PERK-eIF2α-ATF4-CHOP pathway [46, 257] - inhibition of KEAP1-Nrf2/ARE pathway [263] - alteration of miR-34a-5p/Sirt1axis [280] - alteration of the Gpx4/Ager/p65 axis [265, 271]	- ROS-NLRP3-Caspase-1 [58] - p38 MAPK- NF-κB p65-leading to elevation of proinflammatory cytokines [59] - IRE1α/XBP1(ER stress)-NLRP3 [269]
Hg	- GPX4 downregulation [271]	- in LPS treatment Hg disrupts ROS production and inhibits the ASC pyroptosome and GDSMD cleavage
Pb	- GPX4, SLC7a11, Tfrc, and Slc40a1 alterations [274]	- Pb increases NLRP3 expression and Caspase-1 cleavage [53]
U	- increased iron levels and decrease in GPX activity [277] - gamma radiation alters the levels of TRFC, SLC3A2, FTH1, ACSL4, GPX4 [278] - gamma radiation itself generates ROS [279]	no data

## Data Availability

No datasets were generated or analysed during the current study.

## Abbreviations

A20: Tumor necrosis factor, alpha-induced protein 3
AIM: Absent in melanoma
ACSL: Acyl-CoA synthetase long-chain family member
ALA: α-lipoic acid
ALT: Alanine transaminase
AKI: Acute kidney injury
AKT: Protein kinase B
ARE: Antioxidant response element
ASC: Apoptosis associated speck-like protein containing a CARD
AST: Aspartate transaminase
ATG(s): Autophagy related proteins
BAK: B-cell lymphoma 2-antagonist killer 1
BAX: B-cell lymphoma 2-like protein 4
BCSFB: Blood-cerebrospinal fluid barrier
CAPN: Calpain
cGAS-STING: Cyclic GMP-AMP synthase-stimulator of the interferon gene
cIAP: Cell inhibitor of apoptosis protein
Cis-Pt: Cisplatin
CoP(s): Cobalt-chromium-molybdenum implant wear particle(s)
CP: Choroid plexus
CTR: Cu transporter
DAMP(s): Damage associated molecular pattern(s)
DNA: Deoxy-ribonucleic acid
DMT: Divalent metal transporter
ER: Endoplasmic reticulum
ERK: Extracellular signal-regulated kinase
FGF: Fibroblast growth factor
FSP: Ferroptosis suppressor protein
FTL: Ferritin light chain
FTH: Ferritin heavy chain
GPX: Glutathione peroxidase
GSDM(s): Gasdermin(s)
GSH: Glutathione
HMGB: High-mobility group box
HO: Heme oxygenase
HMOX1: Heme oxygenase 1
HRI: Hypoxia-reoxygenation injury
HRG: Heme responsive gene
HSPB1: Phosphorylated Heat shock protein 1
IL: Interleukine
JNK: C-Jun N-terminal kinase
LDH: Lactate dehydrogenase
LOX: Lipoxygenase
LPS-ATP: Lipopolysaccharide-adenosine triphosphate
Lob-Pt: Lobaplatin
KEAP: Kelch-like ECH-associated protein
MAPK: Mitogen-activated protein kinase
mARC: Mitochondrial amidoxime-reducing component
MDA: Malondialadehyde
MEG3: Long non-coding RNA maternally expressed gene 3
miR: MicroRNA
mRNA: Messenger ribonucleic acid
MT: Metallothionein
NCOA: Nuclear receptor coactivator
NEK: NIMA-related kinase
NF-κB: Nuclear factor kappa-light-chain-enhancer of activated B cells
NFR: Nuclear factor erythroid 2–related factor
NLR: Nod like receptor
NLRC: NLR family CARD domain containing protein
NLRP: NLR family pyrin domain containing
PAMP(s): Pathogen associated molecular pattern(s)
PI3K: Phosphoinositide 3-kinase
PUFA(s): Polyunsaturated fatty acid(s)
PTEN: Phosphatase and tensin homolog
Pt-B: Platin B
RNA: Ribonucleic acid
ROS: Reactive oxygen species
Sec: Selenocysteine
SelM: Selenoprotein M
SOD: Superoxide dismutase
S. Typhimurium: Emphasis Type="Italic”>- *Salmonella enterica*</Emphasis> serovar Typhimurium
TFAP2c: Transcription factor AP-2 gamma
TFR: Transferrin receptor
TNBC: Triple-negative breast cancer
TNF: Tumour necrosis factor
TM(s): Transition metal(s)
TRIT: Isopentenyltransferase
TRPC: Canonical transient receptor potential
TXNRD: Thioredoxin reductase
ZEB: Zinc finger E-Box binding homeobox
ZIP: Zrt- and Irt-like proteins
ZnT: Zinc transporter
